# Supplementary material for: Are carbon emissions trading and green financial instruments synergistic? -Comprehensive quantitative research based on content analysis
Source: PLoS One. 2024 Mar 7;19(3):e0298601. doi: 10.1371/journal.pone.0298601 (PMC10919601; doi:10.1371/journal.pone.0298601)
Supplement: S1 Data — (ZIP) [file pone.0298601.s001.zip › Data/地级市Z_F工作报告.zip!/║■▒▒/╗╞╕╘╩╨/└┤╫╘╣1⁄2╓┌║┼í╛┬φ┐╦╩2╛▌═°í┐_2015.docx]

各位代表：

现在，我代表县人民政府向大会报告工作，请予审议，并请县政协各位委员和列席人员提出意见。

一、2014年工作回顾

过去的一年，在县委正确领导下，在县人大和县政协监督支持下，县政府团结和依靠全县人民，突出“双强双兴”，开展“三大行动”， 实施“三大战略” ，统筹稳增长、调结构、促改革、惠民生，经济社会持续稳定健康发展。全县地区生产总值增长10.4%，固定资产投资增长20.7%，规模以上工业增加值增长10.5%，社会消费品零售总额增长13%，外贸出口增长29.7%，公共预算收入增长20.6%，城镇居民和农村居民人均可支配收入分别增长10.02%和12.57%。

（一）转型升级凸现新亮点。工业实现“四升”。产业升级。全县新增规上工业企业19家，产值过亿元企业达到83家。香江电器产值翻番，李时珍医药集团持续增长，未来钢构向健康产业转型。新增医药企业16家，医药产业首位度提高。产品升级。注册商标、精品名牌数量全市第一。设备升级。实施11个技改项目，新增3家“两化”融合试点企业，提高装备水平和自动化程度。技术升级。李时珍医药工业园区成功创建省级高新技术园区，规模以上工业总产值突破百亿元大关。全县新增高新技术企业3家，高新技术增加值增长33.01%。农业呈现“四新”。新基地，粮食产量“十一连增”，总产量稳定在10亿斤以上。新增中药材种植6.38万亩，建成蕲艾、栀子6个万亩连片基地。新增畜禽规模养殖场103个，规模化生产比重72%。水产养殖超过20万亩，建成部级健康养殖示范场6个。全县“三品一标” 企业41家，国家地理标志产品总量全市第一。新企业，发展蕲艾生产企业97家，产值3.97亿元。新增市级龙头企业3家。农产品加工产值占农业总产值比重1.67:1。新模式，通过与电商合作、会员制等方式，农产品电子商务年销售额过亿元。新主体，新增农民专业合作社102家，家庭农场56户。服务业发展加快。完成旅游总体规划编制，推进29个旅游项目建设。三江旅游度假区被评为省级生态旅游示范区，龙泉花海成为3A级景区，李山村被命名为全省旅游名村。开发“百元一日游蕲春”等旅游产品和线路，发展农家乐115家。全年接待游客162万人次，旅游综合收入突破10亿元。麒天下物流园、农产品批发市场开工建设，新建农村综合服务社15家。建成全市首家电子商务孵化园，入驻企业36家。建筑业发展加快，房地产预售面积72万平方米。

（二）“三大行动”取得新成效。开展项目建设大行动。组织重大项目开工月活动。3000万元以上在建项目250个，其中亿元项目113个，完成投资103亿元。扎实开展“三争”，争取普惠性资金15.3亿元，竞争性资金12.6亿元。交通投入9.04亿元，绕城一级公路河西段路基桥涵工程完工，麻武高速进入路面施工，沿江一级公路完成征地拆迁，蕲河白池大桥主跨合龙，新建通村公路127公里。水利投入3.7亿元，水库除险加固、小农水重点县等项目顺利实施。开展招商大行动。引进5000万以上项目78个，其中亿元项目52个，过5亿元项目12个。蕲艾产业园、东风商用车4S店一批重大项目相继落户。开展服务企业大行动。完善五联责任制，推行0713工程，组织融资服务、用工服务、人才培训、评议科股长、电视问政、行风热线、企业与部门面对面等系列活动，开展中介组织专项整治，发展环境有所好转。

（三）统筹城乡渐入新佳境。深入开展“三城联创”，先后荣获省级卫生城、园林城、文明城。启动“多规合一”全域规划，完善中心城区、城南新城、河西新区总规和控制性详规，修编12个乡镇总体规划和2个文化小镇规划，形成总规、控规、详规、专项规划层级完备的规划体系。坚持产城一体，同步推进新城、河西和老城建设。城南新城东璧大道全线贯通，梅家塘大桥完成主体工程，体育路延伸段、十里河东段全部刷黑，“四馆一中心” 开工建设。河西新区“三纵四横”道路框架基本成型。雷溪河旧城改造四大板块同时启动。漕河一路、南阳三路延伸段、京九大道改造全面动工，人民广场完成主体工程，市民广场建成开放，濒湖公园二期抓紧建设。开展乡镇“四个一”工程竞赛，加强农村“五沿”整治，蕲州、横车、刘河成为全国重点镇，龙泉花海、食真公社、赤龙湖风情小镇入围全市重点文化小镇，向桥狮子堰村入选中国传统村落保护名录，横车九棵松、蕲州雨湖被评为省级宜居村庄。深入持久开展“两违”查处，拆除违法建筑3.9万平方米。组织交通拥堵、垃圾围城、占道经营、渣土扬尘等专项整治，建设数字城管中心，市容环境不断改善。

（四）深化改革激发新活力。推进行政审批制度改革。建立权力清单，再造审批流程，推行“三集中、五个一” 模式，实行行政收费零费制和服务收费打包制。推进农村土地制度改革。开展土地确权试点，推进集体建设用地使用权、宅基地使用权确权登记发证和集体林权制度改革。推进金融改革创新。实施大别山金融工程，启动建行“助保贷”和“助农贷”，农商行挂牌运营，新增贷存比同比提高20个百分点，农村金融服务实现全覆盖。连续八届获得“湖北省金融信用县”。推进商事制度改革。工商注册登记实行“先照后证”，各类市场主体增幅全市第一。推进投融资体制改革。依托国建公司融资11.6亿元，组建交投公司，开发区、湿地公园成立投资公司，8家企业挂牌上柜。统筹整合涉农专项资金5.08亿元，发挥规模效应。推进要素保障改革创新。盘活存量土地，处置低效用地，新增建设用地5153亩。坚持安置先行，开工建设还建房3696套。探索政府主体、社区实施拆迁模式，实行村级留地安置，保障村级集体和失地农民长远生计。开展“春风行动” ，组织对口培训、组团招工，帮助企业招工6197人。推进创业创新。建立大学生创业孵化基地，开展大学生实习实训。新增6个校企合作项目，共建研发中心3个，取得省级鉴定科技新成果4项。扩大对外开放。实际利用外资400万美元，新增外贸出口企业4家，香江电器、美春公司、美凯化工成为全市出口大户。

（五）绿色发展取得新突破。推进节能减排。深化陶瓷产业污染综合整治，限制批复高能耗制造业，开展新建小区生活污水治理试点，规上工业企业综合能耗下降5.35%。深化石材整治。开展石材矿山和加工企业集中整治回头看，清理废渣废料，恢复红色旅游公路沿线景观。实施“绿满蕲春”行动。完成人工造林4.5万亩，荣获全省绿化模范县，李山村、龙泉庵村评定为“全省绿色示范村”，仙人湖成为省级示范湿地公园。严格水源保护。开展水库湖泊投肥养鱼和整治禽畜养殖专项治理，重点湖库水质明显好转。整治农村污染。引入市场机制，建立户集村收、乡镇中转、县集中处理的运转体系，启动6个乡镇垃圾处理一体化试点。清洁能源入户新增5000户。

（六）民生保障取得新成绩。大力构建公共服务体系。建成乡镇公办幼儿园15所，改造薄弱学校71所，新实小和特校建成投入使用。通过义务教育基本均衡县国家督导评估。启动公立医院综合改革，基本药物实现网上采购。组建医疗纠纷第三方调解机制，有效化解医患矛盾。全面实施“单独两孩”，严厉打击“两非”，开展孕情全程服务，符合政策生育率稳步上升。深入实施文化惠民工程，李时珍医道文化旅游区被评为全省十大特色文化品牌，非遗保护项目“蕲春艾灸”在俄罗斯参展。再度荣获全国文化先进县。不断完善社会保障体系。公共财政预算收入和税收收入突破10亿元大关，成为全市4个双过10亿元县市之一。民生支出占公共财政预算支出78.2%。全面落实创业就业扶持政策，加大就业培训力度，城镇登记失业率3.98%。企业退休人员养老金“十连调”，建立建筑领域工资支付保障金制度。全面落实五保省级供养标准，开展居家养老服务，开通运行12349居家养老信息平台，80岁以上老人享受高龄津贴。加强残疾人康复、就业、救助，创建全国白内障无障碍县。推进“三位一体”扶贫，减少贫困人口2万人。建设各类保障性住房3231套，中等偏下和低收入住房困难家庭住房保障比重28.8%，改造农村危房1812户。年初承诺的民生实事全面完成。切实加强社会治理。开展平安建设百日会战，打击各类违法犯罪，人民群众安全感不断提高。完成食品药品监管体制改革，建成动物无害化处理中心，漕河四路餐饮一条街和12家餐饮企业被评为全省餐饮服务食品安全示范单位。严格落实安全生产责任制，强化重点领域安全监管，开展打非治违，安全生产保持平稳态势。健全地质灾害、地震应急等体系，提高应急管理水平。深入推进法治蕲春建设，加强社区矫正，健全调处机制。落实信访责任制，畅通信访渠道，积极回应群众诉求，社会大局和谐稳定。

（七）自身建设取得新进步。深入开展党的群众路线教育实践活动，聚焦“四风”，加强突出问题整改，健全联系服务群众“最后一公里”长效机制。建立大督查机制，严格效能问责、治庸问责，政府执行力有所提高。完善“三重一大”决策制度，坚持规范性文件备案审查。积极回应人大代表、政协委员对社会热点问题的关注，办理人大议案建议和政协提案246件。落实党风廉政建设主体责任和“一岗双责” ，推进预防腐败体系建设，认真执行《党政机关厉行节约反对浪费条例》，全面公开部门预算和“三公” 经费。强化行政监察和审计监督，加大惩治腐败力度，查处了一批政府机关工作人员腐败案件。

国防动员、人民防空、民兵预备役和双拥、优抚安置等工作进一步加强。统计、民族宗教、外事侨务、物价、气象、农机、粮食、烟草、县志、档案、保密等工作都取得了新成绩。

各位代表，以上成绩是在全国经济普遍放缓，农产品价格持续低迷，要素制约突出，宏观调控不断加力的背景下取得的，来之不易，难能可贵。这是县委坚强领导、科学决策的结果，是县人大、县政协加强监督、大力支持的结果，是全县上下攻坚克难、共同奋斗的结果！在此，我代表县人民政府，向全县广大干部群众和人大代表、政协委员，向所有参与、关心、支持蕲春发展的同志们、朋友们，表示最衷心的感谢并致以崇高的敬意！

在总结成绩的同时，我们也清醒地看到，全县经济社会发展仍然存在一些突出问题和困难，主要是：当前经济下行压力依然很大，工业投资持续回落，支撑发展的重大项目偏少，稳增长难度加大；制约经济社会发展的矛盾仍然突出，实体经济面临融资难、融资贵等诸多困难，发展的软环境不优，转型发展任务艰巨；基本公共服务尚不健全，财政保障能力不强，涉及人民群众切身利益的住房、出行、教育、医疗、治安、生态环境等与群众期盼还有较大差距；一些公务人员缺乏责任担当，创新能力不强，对新常态不适应、不自觉、跟不上，存在不愿为、不敢为、不会为的问题。我们要主动面对，不回避，不懈怠，积极推进解决。

二、2015年政府工作安排

2015年是全面深化改革的关键之年，全面推进依法治国的开局之年， “十二五”规划的收官之年。做好今年政府工作，必须准确把握新常态，找准工作结合点和着力点，在抢抓机遇、科学发展中把握工作的主动权。要适应经济增长速度换档、结构优化、动力转换影响的新常态，在前进道路上迎接新挑战。要适应国家更加注重中西部地区协调发展和推动长江经济带战略的新常态，在全面对接上抢抓新机遇。要适应稳增长与调结构互动平衡、促进产业转型与市场主体发展双向选择、民生质量与生态质量双重提升的新常态，在跨越赶超上奋力新作为。

今年政府工作的总体要求是：全面贯彻落实党的十八大、十八届三中、四中全会和中央经济工作会议精神，坚持稳中求进的总基调和竞进提质、升级增效的总要求，坚持市场决定取舍、绿色决定生死、民生决定目的三维纲要，主动适应经济发展新常态，把改革创新贯穿于经济社会发展的全过程，着力激发市场活力，着力调整经济结构，着力扩大对外开放，着力保障和改善民生，以求真务实、只争朝夕的进取精神和奋发有为的工作状态，努力开创蕲春科学发展、跨越发展的新局面。

全县经济社会发展主要预期目标是：地区生产总值增长9.5%以上，规模以上工业增加值增长13%以上，固定资产投资增长19%以上，社会消费品零售总额增长13%以上，公共财政预算收入增长15%以上，外贸出口增长12%以上，城镇居民人均可支配收入增长10%以上，农村居民人均可支配收入增长11%以上，城镇登记失业率控制在4%以内，全面完成市定节能减排任务。

围绕实现上述目标，重点抓好五个方面的工作：

（一）坚持竞进作为，推动跨越发展

面对做大总量、提升质量的双重任务，面对广大人民群众对美好生活的新期待，坐是等不来的，坐而论道也是论不来的，必须以竞进的精神状态真干实做，克难奋进。

着力项目建设。更加注重有效投资，补基础设施短板，强产业发展底盘，培植新的经济增长点。精心谋划项目。研究国家政策导向和投资方向，挖掘政策优势，认真编制“十三五”规划。突出抓好基础设施、重大产业和民生项目，扎实推进京九客专、黄冈蕲春机场、大别山火电厂等项目前期工作，争取更多项目进入笼子。抢抓中央密集出台政策，更加重视向中西部倾斜的有利时机，大力开展“三争”，争取更多份额。全力招商引资。完善专班招商、一包到底、绿色通道、督办督导、重奖重惩机制。正确处理数量与质量的关系，提高准入门槛，严格投资强度、税收额度、链条长度、环保程度和科技高度标准，提高招商引资质效。开展签约项目清理，完善奖惩结算和退出机制，切实提高履约率。继续实行产业链招商、专业团队招商、以商引商，力求招商引资大突破。加快项目开工建设。坚持开工是硬道理。把新开工以及续建项目作为重点，开展重大项目开工月活动，一对一服务，强化包保责任，挂图作战，月月督办，公示监督，确保重大项目按期投产。力争新开工亿元项目80个，在建亿元项目170个。

着力产业转型。加快转变农业发展方式。坚持“两转四用” ，不断提高土地产出率、资源利用率、劳动生产率。发展规模经营。加快“五个大县”建设，引导和规范土地经营权有序流转，提升农业规模效益。培育新型农业经营主体。发展农村专业合作社100个，家庭农场50个。引进工商资本建设现代农业示范园，确保宝迪现代农业产业园、万头奶牛产业园和正大淡水渔业生物工程项目建成投产。推进农业产业化经营。加快省级农产品加工园区建设，壮大龙头企业，培植农副产品加工支柱产业。推进农产品品牌化。开展高产创建，推广再生稻“一种两收”吨粮技术，确保粮食稳定在10亿斤以上。推行水产品生态健康养殖、稻田综合养殖和四季捕捞模式，争创农业部渔业健康养殖示范县。建设70个标准化养殖场，创建国家无规定动物疫病区。大力发展“三品一标”，推行清洁生产，发展有机农业、生态农业。做大做强工业。坚持工业立县、工业强县不动摇，把“产业第一，企业家老大”落到实处。实施项目满园工程。增量项目抓开工，存量项目抓进度，储备项目抓前期，形成储备一批、开工一批、投产一批的良性循环。实施企业成长工程。用好大别山产业发展基金、县域经济调度资金、农产品加工园区调度资金，支持李时珍医药集团、香江电器、孺子牛等企业裂变发展，扩规提质。引导“个转企”、“小进规” ，力争新增规模企业15家，亿元企业18家。实施转型升级工程。扶持新兴产业，加强传统产业技术和装备改造。实施质量强县战略，开展“质量兴企”活动，推行卓越绩效管理模式，引导企业从“做产品”向“做品牌”转变，争创驰名商标1个，著名商标4个。完善企业上市培育机制，争取10家企业上柜上市。实施科技创新工程。加快高新技术园区建设，搭建产学研平台，创建高新技术企业，提升核心竞争力。加快推进“两化”融合，培育一批示范企业。加快发展服务业。突破性发展电子商务。加快电子商务孵化园建设，开拓农村电子商务市场。大力发展现代物流，编制物流发展规划，加快管窑物流综合码头、麒天下物流园建设，确保民生广场、农产品批发市场、汽车交易市场建成使用。发展速递配送、冷链储运，构建现代物流体系。突破性发展新型业态。加快推进中介、信息、文化、社区服务业发展。改造传统服务业。加强传统商业模式创新，推动线下与线上、实体店与互联网、城市卖场与社区便民店互动连锁经营。支持房地产和建筑业健康发展。

着力改革创新。推进金融体制改革。大力实施大别山金融工程，加强政银企对接，创新助贷型信贷产品。推进社会信用体系建设，优化金融生态环境。落实金融机构考评奖励和存贷挂钩政策，确保余额存贷比提高5个百分点以上。推进财税体制改革。实行全口径综合预算，建立规范合理的政府债务管理及风险预警机制。完善财政支出结构，推进全过程绩效管理。规范税收秩序，健全综合治税机制，确保税收稳定增长。推进经济领域改革。继续实施市场主体增量提质工程，全面推进先照后证和“三证联办”，力争新增市场主体1万户。加强资金、土地、水、电、气、用工等生产调度，强化要素保障供给。做强国建公司、交投公司及担保公司，逐步实现融资平台市场化、实体化。大力推行PPP模式，拓宽融资渠道。改革财政资金投入机制，统筹专项资金，发挥引导、放大作用。推进农村土地制度改革。通过确权颁证推动土地资源资产化、产权化，引导和规范农村集体经营性建设用地入市，建立农村“三资” 、土地流转、产权交易平台，让农村土地承包经营权、建设用地使用权可流转、可抵押，创造有利于现代农业发展的制度环境。

（二）坚持药旅联动，打造特色品牌

实施医药养生千亿产业工程。充分发掘李时珍品牌价值，打造药材种植、医药加工、医药物流、医药文化、养生旅游全产业链。以药材种植为基础，建立“公司牵头建基地、协会搭桥联农户、乡镇配合抓面积、县里适当拿补贴”的运行机制，突出蕲艾等道地品种，切实抓好沿江、沿河、沿路、沿湖药材长廊建设，新建药材基地5万亩。以医药加工为龙头，引导医药产业向上下游延伸，重点建设蕲艾产业园、李时珍医药集团三期等亿元项目，加快宏中药业、时珍本草、地道中药材、赤方蕲艾等新型加工企业发展，形成企业集群和产业集聚。以健康养生为主题，加快景区建设，开发李时珍纪念馆、蕲州古城等文化资源，运用市场化手段办好李时珍医药节会、全国汽车场地越野锦标赛。以医药物流为引领，加快李时珍国际医药港各功能区建设，积极争取建立省级中药材质量追溯体系和蕲艾质量检测中心、中药材交易中心，汇集资源要素，实现中药材供需线上化、全国化。

推进融合发展。坚持药旅联动，推动文化、医药、生态、农业、城市、工业与旅游的融合，打造产业新优势。开发农业生态休闲功能，促进农村向景区、农业向服务业、农民向工人转变。精心打造李时珍百草园、康特鹿园、檀林雾云山梯田等一批农业发展成果展示平台，建设一批美丽乡村。加快名人文化、医药文化、医道文化、养生文化建设，推动文化与生态旅游、养生养老服务业结合。打造蕲春特色的文化旅游、文物复制、文化研究、文艺演出、艺术培训等产业，加快发展文化创意、现代传媒等新兴文化业态，培育市场主体，扩大文化消费。让城市多一些“药味”、多一些休闲功能，培育更多商业、工业旅游亮点。

突破性发展旅游业。着力彰显“大爱李时珍，养生到蕲春”的特色形象。以“养生蕲春、户外天堂”为引爆点，高标准规划建设李时珍文化旅游区，开发李时珍主题公园、赤龙湖养生养老项目，加快屏风寨、云丹山、医道文化等重点景区建设。开发蕲春药膳，制定特色标准。加快星级酒店建设，大力发展农家乐，努力提高接待能力和水平。开展景区创建，力争创AAAA景区1个，AAA景区2个。建设游客接待中心，设立智慧旅游平台。坚持分工协作、整体营销，建立旅游电子商务，创新多层次营销方式。

（三）坚持全域推进，共建美丽家园

完善规划体系。坚持规划引导、产业支撑，合理确定主体功能分区，完成多规合一的全域规划，实现城乡规划全覆盖。加快编制城区排水防涝综合规划、燃气等专项规划。坚持先骨架后填充，先地下后地上，先重点后一般，统筹抓好县城、中心镇、文化小镇、美丽乡村规划。

坚持产城融合。统筹城区与园区、新城与老城发展。新城抓建设。东璧大道和梅家塘大桥全线刷黑通车，完成绿化景观带和供水管网建设。拉通十里河路、漕河一路、南阳三路至走竹路，打通新老城区路网，实现无缝对接。加快“四馆一中心”和李时珍中医院、老年大学建设，确保主体工程完工。老城抓更新。改造京九大道，延伸付畈大道至沿河路。开工建设财富新天地、义乌商贸城、泰和广场，加快推进城区“生命通道”、“学区通道”和雷溪河环境整治。园区抓配套。加快河西新区基础设施建设，“三纵四横”建成通车，自来水厂实现通水，完成变电站主体工程，启动医院、学校、商贸区和经一路等6条道路建设，做到区域路网贯通，承载功能完善。

推进城乡一体。以改善城乡环境和提高城乡居民生活品质为核心，大力开展洁美乡村、垃圾处理一体化、“三万”、新一轮“三城联创”活动，创建一批省级卫生乡镇、生态乡镇和美丽乡村、卫生乡村。坚持基础设施和公共服务“两个先行”，推进城乡要素平等交换和公共资源均衡配置。全面放开城镇和县城落户限制，引导和支持工业向园区集中、农民向城区集中、土地向大户集中，实现农村人口就地市民化、城镇化。支持蕲州经济发达镇和横车、刘河中心镇建设，做实产业，彰显特色。加快龙泉花海、食真公社等文化小镇建设，发挥示范引领作用，让农村成为安居乐业的美丽家园！

加强城镇管理。完善网格管理，落实“门前四包”、“庭院三包”，加强数字城管建设。严格规划管控，未经规划不建设，未经审批不开工，始终保持高压态势，坚决查处“两违”，切实维护规划的严肃性。组织交通拥堵、沿街为市、垃圾围城、绿化缺失、户外广告、市容环境专项整治，推进城管会战常态化、制度化。延伸城市管理，覆盖新城区规划范围，打造一体化管理平台。完善乡镇规划建设和管理机构，健全机制，提高乡村管理水平。

（四）坚持民生为本，提升幸福指数

建设生态文明。深入实施“绿满蕲春”行动。以争创全省生态文明县为抓手，加强山体、林地、湿地和野生动植物资源保护，推进退耕还林、城镇增绿、“三边”植树工程，大力创建绿色乡村、企业、机关和森林城镇。巩固矿山、石材整治成果，恢复矿山植被。加强节能减排。限制高能耗、高污染项目，淘汰落后产能，推进资源节约和循环利用。开展水源地畜禽养殖、湖库投肥养鱼、石英砂污染河流、陶瓷企业违法排污、城镇集中居民区生活污水重点整治。狠抓环境综合治理。强化工业污染防治，严厉查处违法排污行为，对陶瓷、化工等重点企业加强在线监控，督促实施脱硫、除尘、废水治理及废渣综合利用。在中心城区全面禁止或限制燃放烟花爆竹。加强大气污染防治和建设施工废气与噪声超标治理。整治农村面源污染，推广清洁能源入户。

加强民生保障。坚持就业优先，开发就业岗位，加强就业援助，全面落实创业扶持政策，最大限度增加就业容量。落实被征地农民养老保险政策，提高企业退休人员养老待遇，实施机关事业单位工资和养老保险改革。健全社会救助体系，全面建立临时救助制度，开展城乡低保“按标施保”，推动社会救助由“保障生存”向“保障发展”转型。加快棚户区和农村特别是五保户危房改造，扎实推进保障性住房建设，扩大住房公积金覆盖面。加快居家养老服务中心和农村老年人互助照料中心建设，继续发放高龄补贴。深入推进扶贫攻坚，加快实施安全饮水、特色产业增收、扶贫搬迁、整村推进、通村公路、雨露计划等重点惠民解困工程。尤其是要重视极端贫困，实施精准扶贫，尽力阻断贫困代际传递，让贫困家庭子女机会均等，享受更多的教育公平、就业公平。

强化公共服务。加大学校布局调整力度，加快农村薄弱学校改造，规范促进民办教育，全面实施素质教育。实施文体惠民工程，完善文体设施，开展多层次文体活动。全面推进广播电视村村响、户户通工程。深化医药卫生体制改革，加快公立医院改革，巩固基本药物制度，改进群众就医体验，推进基本公共卫生服务均等化。加强计生基层基础工作，综合治理出生人口性别比，稳定低生育水平。切实保障妇女、儿童、未成年人和残疾人权益，支持慈善事业发展，健全法律援助制度。协调发展民族宗教、外事侨务、对台、保密、档案、县志等各项社会事业。

创新社会治理。深入推进法治蕲春建设，加强法制宣传教育，提高全民法治素养。推进平安细胞工程创建，创新立体化社会治安防控体系，依法严密防范和打击刑事犯罪，努力提高人民群众的安全感。完善社会矛盾纠纷排查调处机制，着力解决群众反映强烈的突出问题，把涉法涉诉信访纳入法治化轨道。建立健全“党政同责、一岗双责、齐抓共管”的安全生产责任体系，全面加强安全生产、消防安全、交通安全、校车安全。加强应急管理，提高公共安全管理和防灾救灾减灾能力。争创全国农产品质量安全示范县，深入开展“餐桌污染”专项治理，用更严的监督、更严的处罚、更严的问责确保食品安全。加强重点领域价格监管，确保物价水平总体稳定。加强人武部正规化建设，做好国防动员、国家安全、民兵预备役和人防工作，推进军民融合发展。

民生决定目的，天下大事必作于细。一个时期以来，我们每年提出为民办十件实事，收到积极成效。从今年开始，我们将把办实事纳入日常工作，更加注重从老百姓最关注、最现实、最直接的问题入手，更加注重从老百姓关心的细微之处抓起，努力办更多的实事，让人民群众从身边的细小变化中，切身感受到生活的改善和提高，感受到社会的公平与正义。

（五）坚持依法行政，加强自身建设

加强法治政府建设。切实转变理念，更加注重运用法治思维深化改革、推动发展、化解矛盾、维护稳定。坚持职权法定，推进政府机构、职能、权限、程序、责任法定化。坚持依法决策，严格执行重大行政决策公众参与、专家论证、风险评估、合法性审查、集体讨论决定的法定程序。建立行政行为纠偏机制，实行县政府领导轮流主持、部门主要负责人参加的行政复议委员会案审会制度，通过“以案说法”，增强各级政府及其部门依法行政意识，规范行政行为。推进政务公开，全面公开政府职能、法律依据、实施主体、职责权限、管理流程、监督方式等事项，重点推进财政预算、公共资源配置、社会公益事业建设等领域的政府信息公开。强化权力监督，自觉接受人大及其常委会法律监督和工作监督，自觉接受政协民主监督，主动接受社会公众监督和新闻舆论监督。认真办理人大代表议案、建议和政协提案、建议案。支持审判机关和检察机关依法独立行使审判权和检察权。

加强服务型政府建设。以“大爱蕲春、大干蕲春”主题活动为载体，更加注重倾听基层群众意见，建立社情民意面对面平台，直接听取市场主体、基层干部、普通群众代表意见，不断改进政府工作。运用群众常用的信息工具，加强政府政策宣传，让政策更及时、更便捷地到达广大群众。全面建立权力清单、责任清单、负面清单，在简政放权、解决“审批多”的基础上，全面实行“三集中、五个一”，着力规范和改进行政审批行为，治理“审批难”。推行“一口受理”，列明审批事项的申请条件、基本流程、示范文本，统一受理，不让基层、企业和群众多跑路。推行“规范办理”，制定审批事项工作细则，明确审查内容、要点和标准，严禁擅自抬高或降低审批门槛，规范自由裁量权。推行“透明办理”，除涉密等不宜公开事项外，公开审批事项的受理、进展、结果信息，杜绝暗箱操作。推行“网上办理”，整合政务服务网络，促进信息资源共享，让审批更加便民、便捷。推行“限时办理”，建立受理单、并联审批和办理时限承诺制度，防止审批事项久拖不决，建设流程规范、服务高效、审批提速的政务服务体系。强化目标管理，完善责任体系，落实清单管理，加强督查督办，实行销号结帐，以钉钉子精神抓落实。严格治庸问责，坚决查处“乱作为”，大力治理“不作为”，着力解决“不会为”，确保令行禁止、政令畅通，提高政府执行力和公信力。

加强廉洁政府建设。严格落实政府系统党风廉政建设的主体责任，切实履行“一岗双责”。加强权力制约，强化财政资金分配使用、国有资产监管、政府投资、政府采购、公共资源转让、公共工程建设等部门内部流程控制，严格行政监察、审计监督和财政监管。严格执行中央八项规定，巩固党的群众路线教育活动成果，扎实开展“三严三实”和尊法纪、讲规矩、有作为教育，继续开展“电视问政”，加强“三公”经费监管，严防“四风”问题反弹，推进作风建设常态化。坚定不移地惩治腐败，提高拒腐防变和抵御风险能力，加大违纪违规案件查处力度，坚决纠正损害群众利益的各种不正之风。

各位代表，时代赋予重托，目标催人奋进！同步奔小康、共筑蕲春梦，是时代赋予我们的光荣使命，是百万蕲春人民的殷切期盼，更是我们职责所系。让我们在县委的坚强领导下，同心同德，众志成城，勇于担当，跨越奋进，以更加昂扬的斗志，以更加生动的实践，为开创蕲春美好未来而努力奋斗！
